# Comparison of Isotope Mass Balance and AquaCrop Model in Evapotranspiration Partitioning in a Maize Field of North China

**DOI:** 10.3390/plants15132059

**Published:** 2026-07-02

**Authors:** Jingjing Wang, Zixuan Wang, Zixun Chen, Bingsun Wu, Guitong Li, Baoguo Li

**Affiliations:** 1 School of Tropical Agriculture and Forestry, Hainan University, Haikou 570228, China; 2 College of Land Science and Technology, China Agricultural University, Beijing 100193, China; 3 Rubber Research Institute, Chinese Academy of Tropical Agricultural Sciences, Haikou 571101, China; 4 College of Resources and Environmental Sciences, China Agricultural University, Beijing 100193, China; 5 Institute of Agro-Environment and Ecology, Hunan Academy of Agricultural Sciences, Changsha 410125, China

**Keywords:** evapotranspiration partitioning, summer maize, isotope mass balance, AquaCrop model, North China Plain

## Abstract

Understanding evapotranspiration (ET) partitioning into soil evaporation (E) and plant transpiration (T) is crucial for improving agricultural water use efficiency in water-scarce regions. The isotope mass balance (IMB) method and AquaCrop model are two widely used approaches for ET partitioning, yet their comparative performance across different crop growth stages remains poorly characterized. This study systematically compared these two methods using two consecutive years (2012–2013) of field isotopic observations in a summer maize field on the North China Plain, a core maize production area facing severe agricultural water scarcity. Stable isotope analysis showed that the local meteoric water line (LMWL) had a slope lower than the global meteoric water line. The 0–5 cm surface soil water evaporation lines had slopes of 5.84 (2012) and 8.06 (2013), confirming significant evaporative enrichment in the topsoil. Plant water isotopic composition closely resembled that of 40–100 cm deep soil water, indicating limited root uptake from the surface layer. IMB-estimated transpiration ratio (T/ET) exhibited distinct phenological patterns, increasing from 37 to 44% at jointing to a peak of 94–96% at filling, then declining to 84–85% at maturity. The two methods agreed well during filling to maturity (differences of 2–10%), but compared with the IMB method, AquaCrop substantially underestimated T/ET at jointing (0.9% vs. 43.8% in 2013) due to its canopy-cover-based transpiration algorithm. These findings identify the filling stage as the critical water demand period, providing a quantitative reference for precision irrigation management under similar climate and soil conditions.

## 1. Introduction

The issue of water resources is of universal concern to the world, which seriously constrains global economic and social development in the 21st century and may lead to conflicts between countries [1,2]. China’s water scarcity threatens global food security, drawing worldwide attention to China’s water resource issues [3,4]. Currently, China faces severe water resource challenges [5,6,7], especially concerning significant shortages and uneven spatial and temporal distribution. The mismatch between water and land resources directly impacts food security [8,9,10]. The North China Plain is the second-largest plain granary in China [11], but it is also an area where water shortage is relatively concentrated. The lack of water resources severely limits the sustainable development of food production [12,13]. Therefore, studying water movement in the soil–plant–atmosphere continuum (SPAC) of the North China Plain is of great significance.

The source and destination of soil water are two research hotspots in the SPAC system. Stable hydrogen and oxygen isotopes can effectively characterize the source of water. Craig’s study of atmospheric precipitation over North America revealed a global-scale linear relationship between hydrogen isotope (δD) and oxygen isotope (δ^18^O) values, expressed as the global meteoric water line (GMWL): δD = 8δ^18^O + 10 [14,15,16]. Based on the relationship between stable δD and δ^18^O in atmospheric precipitation, a local meteoric water line (LMWL) can be constructed, which not only reflects the differences in atmospheric precipitation processes across regions, but also provides a reference for regional water cycle research [17]. However, there is a bias between GMWL and LMWL [18], and the slope and intercept of LMWL typically reflect changes in many geographic and meteorological factors [19,20]. Therefore, when conducting isotope analysis on local precipitation, it is necessary to draw a local atmospheric precipitation line to analyze the composition and formation reasons of stable hydrogen and oxygen isotopes.

Water resources in agricultural ecosystems are mainly consumed through evapotranspiration (ET) [21,22,23,24]. Quantifying ET is essential for reducing unproductive evaporation and ensuring adequate transpiration for crop growth, thereby enhancing agricultural water use efficiency [25]. The evapotranspiration includes productive transpiration (T) and evaporation (E) from land and water surfaces [26,27,28]. Distinguishing E and T improves our understanding of surface water and energy exchange [29] and is fundamental for studying water transport in the soil–plant–atmosphere continuum (SPAC) as well as for irrigation management [30,31].

Several models exist for partitioning ET, including the isotope mass balance (IMB) and AquaCrop models [32,33,34,35]. The IMB method uses variations in hydrogen and oxygen isotopes during precipitation, soil water uptake, and crop water uptake to partition ET [36]. It does not require weather parameters, only water isotopes, and is widely applied in natural and agricultural ecosystems [37,38,39]. However, IMB provides only interval-based calculations over short periods, lacks temporal continuity, and requires numerous assumptions. The AquaCrop model is a widely used, water-driven FAO crop model that has become a standard tool for simulating crop water use, evapotranspiration partitioning, and yield response to water, particularly in water-scarce regions [40,41,42,43]. It emphasizes canopy development and water productivity, and offers continuous and stage-specific simulation with reliable performance under diverse environmental conditions [44,45,46,47]. Ran et al. [42] showed that AquaCrop’s transpiration algorithm, which heavily relies on canopy cover development, tends to underestimate transpiration during early crop development stages when canopy cover is low, a limitation that has not been quantitatively evaluated against isotope-based field observations in rainfed maize systems. In contrast, the IMB method is based on isotopic mass conservation and provides field-constrained estimates independent of canopy dynamics or model parameters. Given their fundamentally different principles—one process-based and water-driven (AquaCrop), the other observation-based and isotope-constrained (IMB)—comparing the two methods is highly valuable for identifying consistencies, discrepancies, and model uncertainties across growth stages. Zhu et al. [48] and Wei et al. [49] demonstrated that isotope-based and model-based evapotranspiration partitioning approaches can yield divergent results across different crop growth stages, highlighting the critical need for systematic side-by-side comparisons to identify their respective strengths and limitations. However, systematic comparisons of these two distinct approaches remain insufficient, especially across key maize growth stages [48]. Therefore, using both IMB and AquaCrop to partition and compare ET fluxes in summer maize fields is of great significance for identifying suitable methods and improving water management in the North China Plain.

Comparing IMB (tracer-based) and AquaCrop (process-based) is not arbitrary [36,48]. Their fundamentally different principles—one constrained by isotopic mass balance, the other driven by canopy cover and water productivity—allow us to test the consistency of ET partitioning across growth stages [36,42,48]. Divergence reveals model limitations (e.g., AquaCrop’s early-stage underestimation); convergence increases confidence. Hence, this comparison aims not to validate one against the other, but to understand their complementarity [49]. IMB provides isotope-constrained field estimates with low temporal resolution [36]; AquaCrop offers continuous simulation but relies heavily on canopy cover [40,41]. A side-by-side comparison in the same field experiment thus enables evaluation of their strengths and limitations for complementary use in water-limited regions like the North China Plain [42,49].

In the North China Plain, monsoonal influence causes highly uneven precipitation, leading to frequent water deficits during the maize growing season [50,51]. This study was conducted in a summer maize field on the North China Plain over two consecutive growing seasons (2012–2013) with three main objectives. The first objective was to characterize the stable hydrogen and oxygen isotopic compositions of precipitation, soil water and plant water, and to identify soil water evaporation patterns and crop root water uptake depths based on isotopic signatures. The second objective was to partition evapotranspiration into soil evaporation and plant transpiration across all key maize growth stages using the IMB method, and to analyze the phenological dynamics of the transpiration ratio. The third objective was to systematically compare the performance of the IMB method and the calibrated AquaCrop model in evapotranspiration partitioning, identify the critical water demand period for summer maize and provide a quantitative reference for precision irrigation management.

## 2. Materials and Methods

### 2.1. Site Description

The field experiment of this study was located in Shangzhuang Experimental Station of China Agricultural University, Shangzhuang Town, northwest Haidian District, Beijing (116°10′53′′ E, 40°08′23.1′′ N, at an elevation of 50 m a.s.l). The Shangzhuang Experimental Station belongs to a warm temperate semi-humid continental monsoon climate, with an average annual precipitation of 595.76 mm and an average temperature of 12.22 °C from 1951 to 2013. The terrain belongs to the alluvial plain in front of the North China Mountains, with a shallow groundwater level. The main soil type is tidal soil, and the soil texture is loam and sandy loam.

### 2.2. Sample Collection

This study collected five main types of data to support evapotranspiration partitioning analysis. These included meteorological data for model input and environmental characterization, stable isotope data of precipitation for hydrological baseline establishment, stable isotope and water content data of soil at multiple depths for water balance calculation, stable isotope data of plant stem water for transpiration flux estimation, and soil temperature data for evaporation isotope calculation. All samples were collected during the five key phenological stages of summer maize to capture seasonal variations in hydrological processes.

The meteorological data of the research area is obtained from the Beijing Station on the China Meteorological Data Sharing Service Network (https://data.cma.cn), with data recorded at 30 min intervals. Precipitation was collected using a custom-made rainwater sampling device, with samples collected immediately after each rainfall cessation and transferred to 100 mL plastic sampling bottles. The bottles were sealed and stored in a refrigerator at 4 °C for preservation.

Soil samples were collected during the jointing, silking, filling, milk ripening, and maturity stages of summer maize using an auger to extract samples from seven soil depths: 0–5, 5–10, 10–20, 20–40, 40–60, 60–80, and 80–100 cm. Each depth was sampled three times. A small portion of each soil sample was placed in aluminum boxes (three replicates) for soil water content determination using the drying method in the laboratory. The remaining soil samples from the same depth were combined into one sample, sealed in plastic bags immediately, and placed in an insulated container to prevent vaporization. These samples were then stored at 4 °C in a refrigerator in the laboratory. Using an R11D2 GIII centrifugal water extraction system (Hitachi, Ltd., Tokyo, Japan), extract the soil water. Set the centrifuge speed to 11,000 rpm, the centrifugation time to 60 min, and the temperature to 4 °C. Each time, 3–5 different plants were sampled, with fresh stems collected, sealed with Parafilm sealing film (Bemis Company, Inc., Neenah, WI, USA), placed in sealed bags, and refrigerated at 4 °C. The stem water was extracted using a low-temperature vacuum distillation system.

The summer maize was sown on June 23 in 2012 and July 1 in 2013, and harvested on October 5 in both years. The total growth periods were approximately 105 days in 2012 and 97 days in 2013. The plants were grown in natural field conditions with calcareous alluvial fluvo-aquic soil as the growth medium, which had a loam to sandy loam texture. The depth of the groundwater table was relatively shallow, ranging from 1.0 to 1.5 m throughout the experimental period. The average daily temperature during the growing season ranged from 22 to 28 °C, with a maximum temperature of 38 °C and a minimum temperature of 15 °C. The plants were grown under rainfed conditions with no artificial irrigation throughout the entire growth period. Basal fertilization was applied before sowing at a rate of 150 kg N ha^−1^, 120 kg P_2_O_5_ ha^−1^, and 90 kg K_2_O ha^−1^, with no additional topdressing during the growing season. The planting density was 60,606 plants per hectare.

Soil temperature measurements were conducted using a T107 soil temperature probe (Campbell Scientific Inc., Logan, UT, USA), with data recorded every 20 min and a measurement error within 0.4 °C. These measurements were used to calculate the oxygen isotope (δ^18^O) and hydrogen isotope (δD) values of the evaporation surface. The determination of hydrogen and oxygen stable isotopes was carried out using a L2120-i water isotope analyzer (Picarro, Inc., Santa Clara, CA, USA), with measurement accuracies of ±0.3‰ (2S.D.) for δ^18^O and ±1‰ (2S.D.) for δD. The results were expressed as the standard per mil difference (δ) relative to the Vienna Standard Mean Ocean Water (VSMOW), according to Equation (1).

(1) $$\delta_{\mathrm{sample}}\;=\frac{R_{\mathrm{sample}}\;-{\;R}_{\mathrm{VSMOW}}}{R_{\mathrm{VSMOW}}}\;\times 1000\;$$

where R refers to the ratio of ^2^H/^1^H or ^18^O/^16^O.

### 2.3. ET Partitioning Model

**Figure 1:**
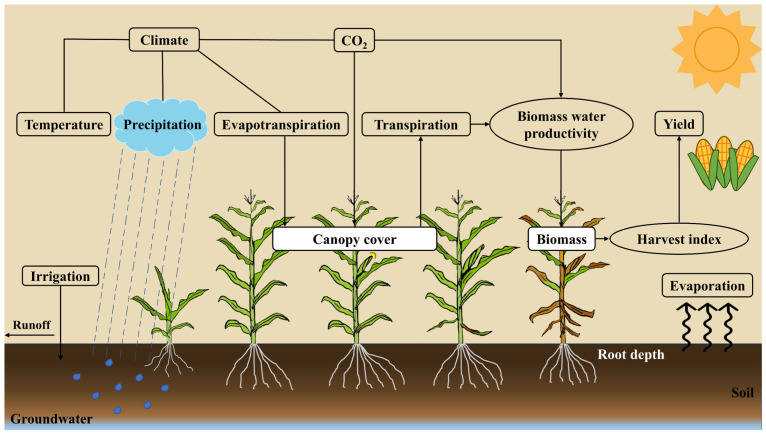
AquaCrop model flowchart adapted for summer maize in the North China Plain.

AquaCrop is a model developed from the theory of maize water growth, which uses soil moisture as the driving factor for crop growth. AquaCrop is developed from the crop water growth theory proposed by Doorenbos and Kassam [52]. AquaCrop employs a simplified canopy growth degradation model to separate evaporation (E) and transpiration (T) in evapotranspiration (ET) (Figure 1). In this study, AquaCrop (Version 6.1) was calibrated and validated for summer maize at the Shangzhuang Experimental Station using field data from 2011 to 2013. Calibration was performed with the 2012 dataset (aboveground biomass, yield, and soil water storage), and validation used the independent 2011 and 2013 datasets, following the procedures described in Hsiao et al. [53] and Heng et al. [54]. Key calibrated crop parameters (Supplementary Table S1 for the complete list) included: normalized biomass water productivity (WP* = 30.70 g·m^−2^), reference harvest index (HI_0_ = 40%), maximum effective rooting depth (1.0 m), base temperature (8 °C), cut-off temperature (30 °C), and crop coefficient when canopy is complete (K_cTr_ = 1.03). Soil hydraulic parameters (residual water content, field capacity, saturated water content, α, n, and saturated hydraulic conductivity) for each soil layer were determined from field measurements (Supplementary Table S1). The model was run under rain-fed conditions (no irrigation), with no fertility or heat stress. The calculation of yield (Y) and biomass (B) was performed according to Equations (2) and (3).

(2) Y=B × HI

(3) B=WP × ∑T

where HI is the harvest index, T is crop transpiration (mm), and WP is biomass water productivity (kg·m^−2^·mm^−1^). The partitioning of evapotranspiration into soil evaporation (E) and transpiration (T) is based on canopy cover development and the Ritchie two-stage evaporation method as implemented in AquaCrop [40,41]. The key input parameters used in the AquaCrop simulations are summarized in Supplementary Table S1, including climate variables, soil profile properties, crop parameters, canopy cover development, rooting depth, phenological dates, and management practices. Model version (6.1) and references for default algorithms [40,41] are provided in the table. Full AquaCrop input parameters are summarized in Supplementary Table S1 with source classification of measured/calibrated/default values.

The isotope mass balance (IMB) model was first proposed by Hsieh et al. [36], primarily based on two mechanisms: water balance and isotope mass balance. The calculation was performed according to Equations (4)–(7).

(4) $$m_{f}-m_{o}\;=m_{p}+{\;m}_{i}\;+m_{u}\;-{\;m}_{r}-m_{d}-m_{e}\;{-m}_{t}$$

(5) $$m_{f}\delta_{f}-m_{o}\delta_{o}={\;m}_{p}\delta_{p}+{\;m}_{i}\delta_{i}\;+m_{u}\delta_{u}-m_{r}\delta_{t}-m_{d}\delta_{d}-m_{e}\delta_{e}-m_{t}\delta_{t}$$

(6) $$m_{\mathrm{ET}}=m_{e}+m_{t}$$

(7) $$\begin{array}{c} m_{e}=\frac{\left\lfloor m_{p}(\delta_{p}-\delta_{t})+m_{i}(\delta_{i}-\delta_{t})+m_{u}(\delta_{u}-{\;\delta }_{t})+{\;\delta }_{t}(m_{f}-m_{o})\right\rfloor }{\delta_{e}-\delta_{t}}\\ -\frac{m_{d}(\delta_{d}-\delta_{t})+m_{r}(\delta_{r}-\delta_{t})+(\delta_{f}-\delta_{o})(m_{f}-m_{o})}{\delta_{e}-\delta_{t}} \end{array}$$

where m_0_ and δ_0_ are the initial water storage capacity (mm) and soil water δ^18^O isotope values (‰) of a certain depth of soil in the study area; m_f_ and δ_f_ are the final water storage capacity (mm) and soil water δ^18^O isotope values (‰) of a 1 m soil mass in the study area; m_p_ and δ_p_ are the precipitation (mm) and δ^18^O isotope values of precipitation in the study area (‰); m_i_ and δ_i_ are the irrigation amount (mm) and the δ^18^O isotope values (‰) of irrigation water in the study area; m_u_ and δ_u_ are the capillary rising water volume (mm) and the δ^18^O isotope values (‰) of capillary rising water in the study area; m_r_ and δ_r_ are the surface runoff (mm) and δ^18^O isotope values (‰) of runoff in the study area; m_d_ and δ_d_ are the amount of soil water leakage in the study area (mm) and the δ^18^O isotope values of soil water leakage (‰); m_e_ and δ_e_ are the soil surface evaporation (mm) and evaporation δ^18^O isotope values (‰) in the study area; m_t_ and δ_t_ represent the plant transpiration (mm) and the δ^18^O isotope values (‰) of plant transpiration water in the study area. The IMB calculations assume: (i) isotope values of transpiration water (δ_t_) equals those in stem water (no fractionation during uptake); (ii) evaporation water (δ_e_) is derived from the Craig–Gordon model using measured soil temperature and humidity; (iii) the isotopic composition of runoff and deep percolation equals that of the corresponding soil water layer; (iv) soil water storage change is accounted for by the term δ_f_ m_f_ − δ_0_ m_0_; and (v) no other vapor exchange (e.g., condensation) occurs. These assumptions are standard [36,48] and necessary to solve the balance equations with limited field data. The derivation of runoff, deep percolation, and upward capillary rise for the IMB calculations (Table 1) is described in Supplementary Text S1. Precipitation, irrigation, and soil water storage change were directly measured as outlined in Section 2.2.

### 2.4. Uncertainty Estimation for IMB-Derived T/ET

Because only one transpiration ratio (T/ET) value was obtained per growth stage from the IMB method, no statistical standard deviation could be calculated from repeated measurements. To account for the combined effects of uncertainties in the input parameters (δ_e_, δ_t_, runoff, deep percolation, capillary rise, and soil water storage change), we adopted a conservative relative uncertainty of ±5% for the T/ET ratio. This value is consistent with typical uncertainties reported in isotope-based evapotranspiration partitioning studies [36,48] and reflects the analytical precision of the isotope analyzer (δ^18^O: ±0.3‰, δD: ±1‰) as well as an assumed ±10–15% uncertainty for the water balance components. The detailed IMB-derived evaporation (E), transpiration (T), and T/ET values with their estimated uncertainties for each growth stage are provided in Supplementary Table S2.

### 2.5. Statistical Analysis

For specific parameters and related data of the AquaCrop model [55]. Statistical analysis was conducted using SPSS software (SPSS for Windows, Version 20.0, International Business Machines Corporation, Chicago, IL, USA). Pearson correlation analysis was employed, with significance levels set at 0.05 and 0.01. Plots were generated using Origin 2021 (OriginLab Corporation, Northampton, MA, USA), Excel 365 (Microsoft Corporation, Redmond, WA, USA), and SigmaPlot 15.0 software (Systat Software Inc., San Jose, CA, USA).

## 3. Results

### 3.1. Atmospheric Precipitation and Characteristics of Stable Isotopic Distribution of Hydrogen and Oxygen in Precipitation

**Figure 2:**
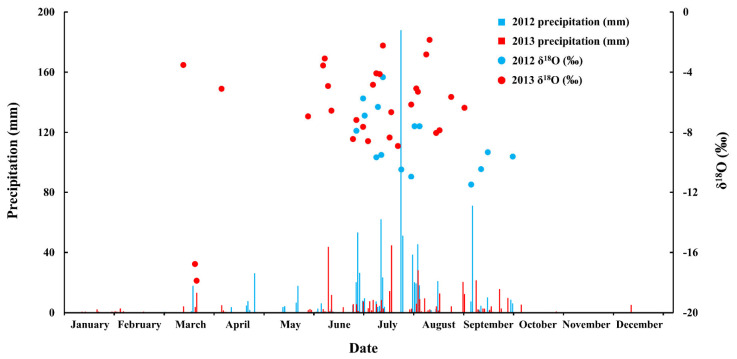
Partitioning of evapotranspiration and oxygen isotope (δ^18^O) in the study site in 2012–2013.

The collected precipitation samples are mostly concentrated from June to September in summer, which happens to be the growing season of summer maize. This study collected a total of 67 precipitation samples from 2012 to 2013 (Figure 2). The value of hydrogen isotope (δD) in the collected atmospheric precipitation ranged from −6.41 to −123.01‰, with an average value of −50.01‰; the oxygen isotope (δ^18^O) ranged from −1.85 to −17.88‰, with an average value of −7.18‰.

### 3.2. Local Meteoric Water Line Equation

Samples of atmospheric precipitation were collected throughout the entirety of 2012–2013. δ^18^O values of atmospheric precipitation ranged from −4.33 to −11.48‰ in 2012, while δ^18^O values of soil water varied from −4.90 to −11.70‰. In 2013, δ^18^O values of atmospheric precipitation ranged from −2.23 to −9.60‰, and δ^18^O values of soil water ranged from −2.83 to −8.77‰. The slopes of the local meteoric water line in 2012 and 2013 were 6.52 and 6.56, respectively, with intercepts of −7.01 and −1.54. The slopes and intercepts were both smaller than those of the global meteoric water line (GMWL).

**Figure 3:**
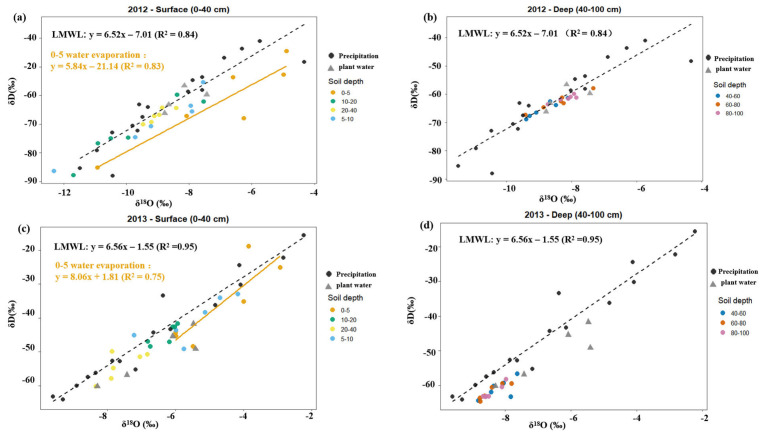
δD–δ^18^O relationships for different water sources in 2012 and 2013. (**a**) 2012 surface soil (0–40 cm), (**b**) 2012 deep soil (40–100 cm), (**c**) 2013 surface soil, (**d**) 2013 deep soil. Soil depths are color-coded (see legend). Black dashed line: local meteoric water line (LMWL) with equation and R^2^ (upper left). Orange solid line in (**a**,**c**): 0–5 cm soil water regression (equation and R^2^ below LMWL). Precipitation: black circles; plant water: gray triangles.

According to the mechanism of stable isotope fractionation in soil water and previous studies [56], soil evaporation mainly occurs in the 0–40 cm surface layer, where hydrogen and oxygen isotopes are strongly enriched by evaporation. In contrast, the 40–100 cm deep layer is barely affected by evaporation, showing stable isotopic compositions. Therefore, we grouped all soil depths into surface soil (0–40 cm) and deep soil (40–100 cm). To quantify evaporative enrichment, we focused on the uppermost 0–5 cm layer and performed linear regression to establish the evaporation line for surface soil water. In 2012, the fitted evaporation line for the 0–5 cm layer was δD = 5.84 δ^18^O − 21.13 (R^2^ = 0.83). Its slope (5.84) was lower than that of the LMWL (6.52), indicating that strong evaporative fractionation mainly occurred in the surface soil layer (Figure 3a,b). In 2013, the corresponding surface soil evaporation line was δD = 8.06 δ^18^O + 1.81 (R^2^ = 0.75). The slope (8.06) was higher than that of the LMWL (6.56), suggesting that additional factors (e.g., mixing with different water sources or higher initial δ^18^O values) may have influenced the isotopic signature of surface soil water in that year. The isotopic relationships of all water sources in 2012 and 2013 are presented in Figure 3, where panels (a) show surface and deep soil water for 2012, and panels (b) correspond to 2013.

### 3.3. Partitioning of Evapotranspiration from Summer Maize Fields

**Figure 4:**
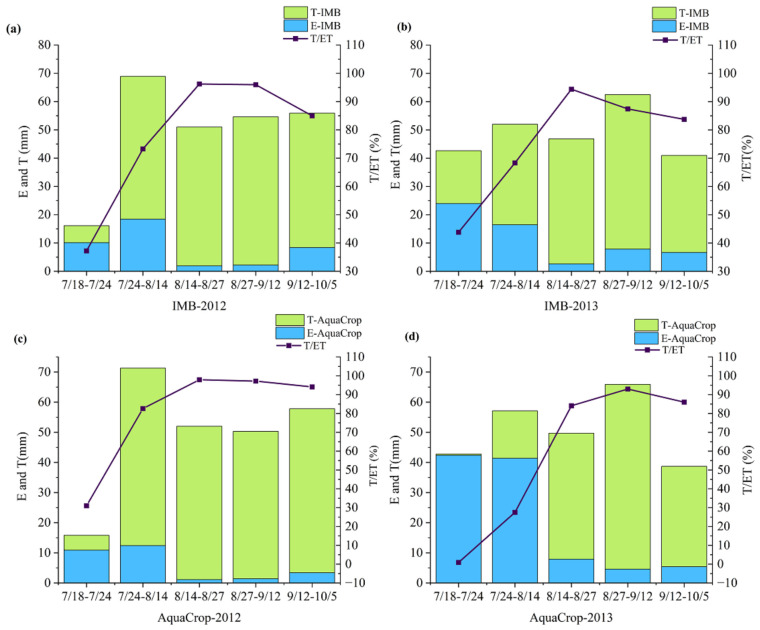
Dual-axis plots of evapotranspiration partitioning for summer maize in the North China Plain based on the IMB model (**a**,**b**) and the AquaCrop model (**c**,**d**) for 2012 and 2013. (**a**) IMB, 2012; (**b**) IMB, 2013; (**c**) AquaCrop, 2012; (**d**) AquaCrop, 2013. Stacked bars represent soil evaporation (E) and plant transpiration (T) (mm), and the line with symbols represents the transpiration ratio (T/ET) (%). Phenological stages from left to right: jointing, tasseling, filling, milk ripening, and maturity.

This study used the isotope mass balance (IMB) method to distinguish the evapotranspiration of summer maize at depths of 0–100 cm in the study area from 2012 to 2013. In 2012, the value of the δ^18^O of transpiration (δ_t_) ranged from −11.16 to −6.64‰, the value of the δ^18^O of evaporation (δ_e_) ranged from −19.25 to −18.33‰ (Table 1), the value of total evapotranspiration ($m_{\mathrm{ET}}$) ranged from 16.10 to 68.90 mm, the value of soil evaporation (m_e_) ranged from 1.92 to 18.43 mm, and the value of plant transpiration (m_t_) ranged from 5.99 to 52.39 mm (Figure 4). In 2013, the value of the δ^18^O of transpiration (δ_t_) ranged from −7.47 to −4.05%, and the value of the δ^18^O of evaporation (δ_e_) ranged from −16.47 to −15.37% (Table 1). The value of $m_{\mathrm{ET}}$ ranges from 40.96 to 62.48 mm, the value of m_e_ ranges from 2.62 to 23.86 mm, and the value of m_t_ ranges from 18.69 to 54.63 mm (Figure 4).

**Figure 5:**
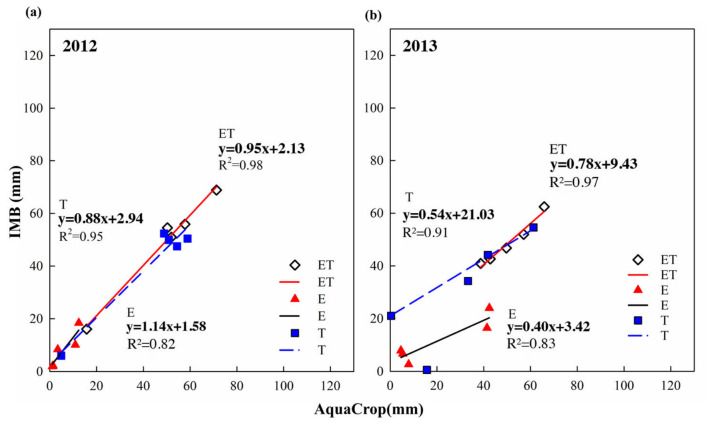
Linear regression analysis of AquaCrop-simulated versus IMB-estimated soil evaporation (E), plant transpiration (T) and total evapotranspiration (ET) for summer maize in 2012 (**a**) and 2013 (**b**). All values are in millimeters (mm). Each point represents the cumulative flux over one phenological stage (n = 5 stages per year). Regression equations and coefficients of determination (R^2^) are displayed in each panel.

Evapotranspiration (ET) from both approaches is quantified via water balance principles, yet they adopt distinct algorithms to partition total ET into soil evaporation (E) and crop transpiration (T). Linear regression was performed to compare ET, E and T estimates derived from the two methods. In 2012, the regression slopes for ET, E and T were 0.95, 1.14 and 0.88, respectively (Figure 5a), whereas corresponding values dropped to 0.78, 0.40 and 0.54 in 2013 (Figure 5b). Overall, the agreement between the two models for ET was higher than for T and E, with E showing the strongest interannual variability in performance.

Coefficient of determination (R^2^) in 2012 reached 0.98 (ET), 0.82 (E) and 0.95 (T) (Figure 5a); in 2013, R^2^ changed to 0.97 (ET), 0.83 (E) and 0.91 (T) (Figure 5b). Specifically, R^2^ of ET and T was higher in 2012 than in 2013, while E showed the opposite trend (higher in 2013 than in 2012). Collectively, regression outputs reveal inconsistent agreement between the IMB and AquaCrop methods across variables and years. Despite high R^2^ demonstrating strong linear correlations, slopes of E (0.40) and T (0.54) were substantially below unity in 2013, pointing to significant proportional bias: AquaCrop severely overestimated E but underestimated T in 2013.

Complementary error statistics, including root mean square error (RMSE, mm), mean absolute error (MAE, mm), mean bias error (bias, mm), mean absolute relative error (RE, %), Nash–Sutcliffe efficiency (NSE) and Willmott’s index of agreement (d) are summarized in Supplementary Table S3. All error indicators reflected favorable consistency in 2012: ET (RMSE = 2.40 mm, NSE = 0.98), E (RMSE = 3.56 mm, NSE = 0.66), T (RMSE = 5.20 mm, NSE = 0.91), with limited systematic bias. By contrast, obvious systematic bias occurred in 2013: AquaCrop markedly overestimated E (RMSE = 14.16 mm, bias = +8.83 mm, RE = 98.1%) and underestimated T (RMSE = 12.48 mm, bias = −6.97 mm, RE = 34.8%). This reciprocal offset between E overestimation and T underestimation results in an acceptable overall ET simulation (NSE = 0.83). Crucially, this systematic proportional bias is fully concealed by the high R^2^ values ranging from 0.83 to 0.97, which highlights that evaluating model consistency solely via R^2^ is inappropriate and misleading.

This study partitioned E and T and their proportions to ET using the IMB and AquaCrop models (Figure 4). The IMB-derived T/ET ratios during the jointing stage were 37.17% (2012) and 43.82% (2013), higher than the AquaCrop values (31.0 and 0.9%, respectively). At tasseling, IMB gave 73.25% (2012) and 68.34% (2013), while AquaCrop gave 82.6 and 27.5%, showing a notable discrepancy in 2013. During filling and milk ripening, both models produced similarly high T/ET ratios (IMB: 94–96%, AquaCrop: 84–98%). At maturity, IMB showed 84.97% (2012) and 83.73% (2013), whereas AquaCrop gave 94.1% and 86.0% (Figure 4a–d).

### 3.4. The Relationship Between ET, E, T and Climatic Environmental Factors

**Figure 6:**
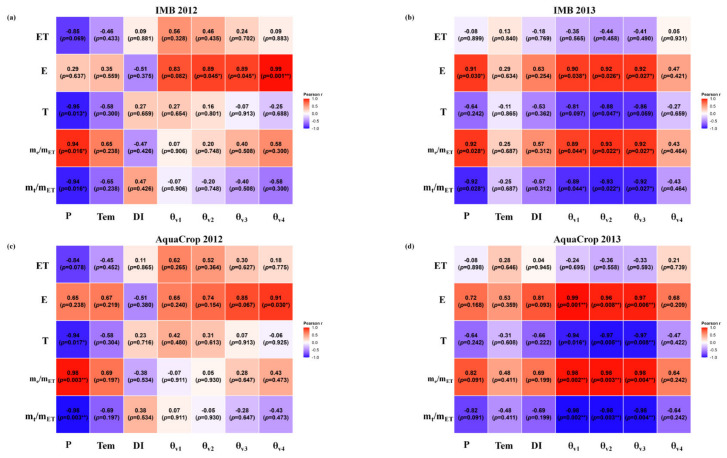
Pearson correlation heatmaps between evapotranspiration (ET), soil evaporation (E), plant transpiration (T), evaporation ratio (m_e_/m_ET_), transpiration ratio (m_t_/m_ET_) and climatic and soil environmental factors. Subplots (**a**,**b**) represent results from the isotope mass balance (IMB) model for 2012 and 2013, respectively; subplots (**c**,**d**) represent results from the AquaCrop model for 2012 and 2013, respectively. P, precipitation; Tem, air temperature; DI, drought index; θ_v1_–θ_v4_, volumetric soil water content at 0–5, 5–10, 10–20 and 20–40 cm, respectively. Asterisks denote statistical significance: * *p* < 0.05, ** *p* < 0.01.

This study analyzed the relationships between evapotranspiration (ET), soil evaporation (E), plant transpiration (T), evaporation ratio (m_e_/m_ET_), transpiration ratio (m_t_/m_ET_) and climatic/environmental variables (precipitation P, air temperature Tem, drought index DI, and soil volumetric water content at 0–5 cm (θ_v1_), 5–10 cm (θ_v2_), 10–20 cm (θ_v3_), and 20–40 cm (θ_v4_) using Pearson correlation analysis (Figure 6). All analyses are based on five growth stages per year (n = 5 for each year); therefore, *p*-values should be interpreted cautiously, and the results are considered exploratory rather than confirmatory.

For the IMB model in 2012 (Figure 6a), T and m_t_/m_ET_ were significantly negatively correlated with P (*p* = 0.013 and *p* = 0.016, respectively). E was significantly positively correlated with θ_v2_ (*p* = 0.045 and *p* = 0.045, respectively), and extremely significantly positively correlated with θ_v4_ (*p* = 0.001). ET showed no significant correlation with any variable. For the AquaCrop model in 2012 (Figure 6c), T was significantly negatively correlated with P (*p* = 0.017). In 2013, IMB results (Figure 6b) showed that E and m_e_/m_ET_ were significantly positively correlated with P (*p* = 0.030 and *p* = 0.028, respectively). Both IMB and AquaCrop models (Figure 6b,d) exhibited positive correlations of E and me/m_ET_ with θ_v_, θ_v2_, and θ_v3_. For IMB outputs, m_t_/m_ET_ was negatively correlated with θ_v1_, θ_v2_, and θ_v3_, and T was negatively correlated with θ_v2_. Similarly, AquaCrop-simulated T and m_t_/m_ET_ were negatively correlated with θ_v1_, θ_v2_, and θ_v3_. Overall, both models showed comparable relationships with soil moisture (positive for E and m_e_/m_ET_, negative for T and m_t_/m_ET_), while correlations with precipitation were less consistent across years and models.

## 4. Discussion

### 4.1. Characteristics of Water Exchange at the Atmospheric Soil Boundary

A key finding of this study is that the local meteoric water line (LMWL) had a lower slope than the global meteoric water line, and the 0–5 cm surface soil water showed strong evaporative enrichment. This study found that δ^18^O in summer precipitation is negatively correlated with precipitation amount, indicating that summer rainfall is predominantly influenced by monsoons, consistent with previous research findings [57,58,59]. The precipitation amount effect (where the isotopic composition in precipitation shows a negative correlation with rainfall amount) is a fundamental characteristic of isotopes in monsoon regions [60,61,62]. This effect is commonly observed in low-latitude oceans and monsoon regions [63,64,65], while in some mid-latitude regions, it may occur only in summer [57]. Compared to the global meteoric water line (GMWL), the slopes and intercepts of the local meteoric water lines (LMWL) were both smaller in 2012 and 2013, consistent with the findings of Hao et al. [66]. This indicates that the precipitation processes in 2012–2013 underwent secondary evaporation effects [67].

As shown in Figure 3, the isotopic signatures of soil water clustered around the LMWL, confirming that soil water primarily originates from atmospheric precipitation recharge. In this study, the slope of the soil water evaporation line in the 0–5 cm soil layer in 2012 was 5.84, which is lower than the local atmospheric precipitation line slope of 6.52. This suggests that there was isotopic enrichment due to evaporative fractionation in the shallow soil surface water, indicating that the proportion of soil water utilized by plant root uptake from the shallow soil layer should be relatively small. This pattern of surface evaporative enrichment is consistent with observations in other monsoon-influenced agricultural soils [66,67].

### 4.2. The Division of Evaporation and Transpiration Fluxes Using the IMB Model

The main finding from the IMB model is the clear phenological pattern of the transpiration ratio (T/ET), which increased from jointing (37–44%) to a peak at filling (94–96%) and then declined to 84–85% at maturity. As presented in Table 1 and Figure 4a–b, evaporation dominated ET during the jointing stage due to low canopy coverage, consistent with findings from other studies [64,65,66]. Correspondingly, transpiration reached its lowest proportion (37–44%) at jointing, attributed to the small leaf area index and short plant stature [49,68,69]. By tasseling, transpiration became the dominant component (68–73% of ET) due to increased canopy coverage [70]. The filling stage exhibited the highest T/ET (94–96%), marking the period of maximum water demand as the leaf area index peaked [71]. T/ET remained high during milk ripening (87–96%), reflecting continued strong photosynthetic activity. At maturity, T/ET declined to 84–85% as plant growth weakened and evaporation became relatively more important [72]. The T/ET values reported here are comparable to those found in other maize fields under similar climatic conditions [73,74], confirming that transpiration dominates total ET during reproductive stages in rain-fed systems. Furthermore, Wang et al. [75] demonstrated that groundwater depth can significantly influence maize transpiration and water productivity in arid areas, which supports our observation that shallow groundwater (1.0–1.5 m) may have contributed to the consistently high T/ET in our study. The global synthesis by Wang et al. [68] also confirmed that vegetation canopy development exerts a dominant control on evapotranspiration partitioning across different ecosystems, aligning with our phenological interpretation.

### 4.3. Differences Between IMB Model and AquaCrop Model in Partitioning Evapotranspiration

In both 2012 and 2013, the transpiration ratio (T/ET) from the two models followed a similar seasonal pattern—increasing from jointing to filling and then slightly decreasing. However, notable discrepancies emerged during the early growth stages: AquaCrop produced substantially lower T/ET values at jointing and tasseling compared to IMB. These observed discrepancies likely arise because AquaCrop computes transpiration based on canopy cover [76], which remains low in early stages and may not fully capture actual transpiration dynamics. This underestimation of early-season transpiration by AquaCrop is consistent with previous evaluations of the model in maize systems [42], further supporting the need for site-specific calibration of canopy cover parameters. In contrast, both models yielded consistent T/ET ratios during filling, milk ripening, and maturity, indicating comparable performance under full canopy conditions. Additionally, Wu et al. [77] reported that machine learning models and process-based models like AquaCrop require careful calibration at early growth stages to avoid underestimation of transpiration.

Beyond the T/ET ratio, regression analysis (Figure 5) revealed obvious interannual divergence in model performance. In 2012, AquaCrop achieved favorable simulation of ET components with regression slopes close to 1 and satisfactory error statistics; by contrast, the model systematically overestimated soil evaporation (E) and underestimated transpiration (T) in 2013, with slopes of only 0.40 (E) and 0.54 (T) against IMB. The low slope for E in 2013 may be explained by: (1) AquaCrop’s simplified evaporation routine, which relies heavily on canopy cover and surface wetness and may not accurately simulate evaporation under the specific rainfall pattern of 2013; (2) potential inaccuracies in input parameters such as soil hydraulic properties and initial soil moisture; and (3) uncertainties inherent to IMB estimates, though these alone are unlikely to account for the consistent low bias. This underestimation of early-season transpiration by AquaCrop is consistent with previous evaluations of the model in maize systems [42], further supporting the need for site-specific calibration of canopy cover parameters. These findings suggest that AquaCrop’s evaporation module would benefit from site-specific calibration, especially for years with moderate rainfall distributions. A slope of 0.40 for E indicates that AquaCrop’s simulated E increases faster than IMB-estimated E, leading to progressive overestimation as actual evaporation increases.

The low regression slopes for 2013 indicate a proportional bias: AquaCrop’s overestimation of E and underestimation of T become more pronounced as the magnitudes increase. This is confirmed by the error metrics in Supplementary Table S3, which show E was severely overestimated (Bias: +8.83 mm, RE = 98.1%) while T was systematically underestimated (Bias: −6.97 mm, RE = 34.8%). In contrast, the high R^2^ values (0.83–0.97) merely reflect strong linear correlation rather than absolute numerical agreement. Therefore, relying solely on R^2^ to judge model consistency would be misleading, and multiple error indicators are required for comprehensive performance evaluation.

The observed discrepancies are consistent with the correlation results (Section 3.4), where the IMB method showed minimal environmental correlations, while AquaCrop exhibited strong links between soil moisture and ET components—reflecting their fundamentally different underlying assumptions (isotope-constrained field observations vs. mechanistic canopy-cover-driven algorithms). Neither method should be regarded as an absolute ground truth; their complementary use offers a more robust understanding of ET partitioning [78]. A limitation of this study is that the calibration of AquaCrop relied on a single-site dataset (Shangzhuang Experimental Station); cross-site validation is needed to improve the generalizability of the calibrated parameters. While this approach ensures transparency and transferability, calibrating key parameters (e.g., CGC, WP*) could improve simulation accuracy, particularly for soil evaporation in years with distinct rainfall patterns (e.g., 2013). Future work should consider calibration using independent field data.

### 4.4. Factors Affecting ET, E, and T

Understanding the controls on evapotranspiration partitioning is essential for refining process-based models and irrigation scheduling. Using Pearson correlation analysis (Figure 6), we examined the relationships of evapotranspiration (ET), soil evaporation (E), plant transpiration (T), evaporation ratio (E/ET), and transpiration ratio (T/ET) with climatic and soil variables for both models in 2012–2013.

Divergent correlation patterns between IMB and AquaCrop. For the IMB model, significant correlations (*p* < 0.05) were found for θ_v2_ vs. θ_v3_ and m_e_/m_ET_ vs. m_t_/m_ET_. Correlations with precipitation were significant only in 2012 and not in 2013, while temperature and soil moisture showed no consistent significant correlations across years. This pattern reflects the integrative nature of isotope-based partitioning: it captures net effects over each growth stage, smoothing short-term hydrological fluctuations. Conversely, AquaCrop showed strong, numerous correlations, especially between soil moisture at multiple depths and ET components. This difference is not coincidental: AquaCrop’s algorithms explicitly link evaporation to surface soil moisture and canopy cover, and transpiration to root-zone water availability [44,45,46,47]. Therefore, its water-driven formulation may overstate the role of soil moisture compared to field reality.

Neither temperature nor drought index showed significant correlations with any ET component in either model. This is consistent with the small intra-seasonal temperature range (monthly means 24.1–24.5 °C, SD 2–3 °C) and the fact that the drought index integrates rainfall and temperature over long periods, which may not align with the stage-specific ET dynamics.

A key insight is that the seasonal increase in T/ET is driven by crop phenology, not by precipitation amount. Despite a marked difference in growing-season rainfall between 2012 (461 mm) and 2013 (261 mm), the T/ET trajectories were nearly identical—rising from jointing to a filling peak and then declining. This demonstrates that the seasonal increase in transpiration ratio is primarily governed by crop phenology (canopy expansion, leaf area index) rather than by precipitation or soil water content [49,68,69]. Soil moisture can modulate transpiration under dry spells, but it does not override the phenological pattern [79,80]. Consequently, the lack of significant precipitation correlations in IMB does not imply that rainfall is unimportant. Instead, its effect is buffered by antecedent soil storage and subsumed by the phenological timetable [81,82,83]. Similarly, the strong correlations in AquaCrop should be interpreted cautiously: they arise partly from the model’s structural dependence on soil water, not necessarily from a dominant environmental control in the field [84].

Implications for model use and water management. These contrasting patterns highlight fundamental differences between the two approaches. IMB, anchored in field-measured isotopic signatures, reflects actual biophysical fluxes but suffers from low temporal resolution and sampling uncertainty [36,48]. AquaCrop provides temporal continuity and mechanistic clarity but may exaggerate the influence of soil moisture because of its simplified routines [40,41,42,43]. Neither method is an absolute ground truth; together, they offer complementary insights [31,77]. From a practical standpoint, our results indicate that irrigation should focus on securing soil moisture during the filling stage—when T/ET exceeds 94%—rather than responding to individual rainfall events, which do not independently govern transpiration at seasonal scales [71,72]. Recent advances in evapotranspiration simulation using machine learning (Wu et al. [77]) also emphasize the importance of capturing stage-specific dynamics, which aligns with our finding that phenology outweighs precipitation in controlling T/ET.

## 5. Conclusions

The North China Plain faces severe agricultural water scarcity despite being a core summer maize production region. Using two consecutive years of field observations, this study quantified evapotranspiration (ET) partitioning into evaporation (E) and transpiration (T) via stable isotopes, and compared the performance of the AquaCrop model and the isotope mass balance (IMB) method. Stable isotope results showed that the local meteoric water line (LMWL) was δD = 6.56δ^18^O − 1.54 (R^2^ = 0.96). The regression slopes for soil water within the 0–5 cm layer were 5.84 in 2012 and 8.06 in 2013, confirming that evaporative fractionation mainly occurred in the topsoil. The isotopic signature of plant water was consistent with that of soil water at 40–100 cm depth, indicating that maize roots took up little moisture from the surface layer. The IMB method revealed a distinct phenological pattern in the transpiration ratio (T/ET). This ratio rose from 37 to 44% at the jointing stage to a peak of 94–96% at the filling stage, and decreased to 84–85% at maturity. The AquaCrop model showed good consistency with IMB results from the filling stage to maturity, with differences ranging from 2 to 10%. However, it substantially underestimated T/ET at the jointing stage, with a simulated value of 0.9% compared with the measured value of 43.8% in 2013. This discrepancy stems from the model’s transpiration calculation based on canopy cover. The filling stage is identified as the critical water demand period for summer maize. Since maize roots primarily absorb water from the 40–100 cm soil layer and surface soil moisture is prone to evaporation, irrigation at this stage should prioritize sustaining water availability within the 40–100 cm root zone. Moderate deep irrigation is recommended instead of frequent shallow irrigation. This study provides a quantitative reference for precision irrigation management for summer maize across areas with similar climate and soil conditions in the North China Plain.

## Figures and Tables

**Table 1:** The soil water balance calculated for the entire summer maize growth period at 0–100 cm by the isotope mass balance in the soil profile with oxygen isotope (δ^18^O) in 2012–2013.

Year	Parameter	Date				
		(18 July–24 July)	(24 July–14 August)	(14 August–27 August)	(27 August–12 September)	(12 September–5 October)
2012	m_f_ − m_0_ (mm)	94.30	−23.20	−65.19	59.81	−56.92
	δ_f_m_f_ − δ_0_m_0_ (‰)	−1342.62	563.50	473.35	−684.74	641.96
	m_p_ (mm)	238.60	126.10	1.30	84.60	10.70
	δ_p_m_p_ (‰)	−2495.76	−1065.39	−14.93	−882.87	−62.75
	m_r_ (mm)	100.30	23.70	0.00	8.10	0.00
	δ_r_m_r_ (‰)	−647.94	−200.24	0.00	−84.53	0.00
	m_d_ (mm)	27.90	56.70	15.50	0.00	11.70
	δ_d_m_d_ (‰)	−268.68	−510.82	−127.22	0.00	−104.71
	m_u_ (mm)	0.00	0.00	0.00	37.90	0.00
	δ_u_m_u_ (‰)	0.00	0.00	0.00	−336.58	0.00
	δ_t_ (‰)	−7.75	−11.16	−6.64	−7.79	−9.25
	δ_e_ (‰)	−18.80	−19.25	−18.33	−19.13	−19.11
	E (mm)	10.12	18.43	1.92	2.20	8.40
	T (mm)	5.99	50.47	49.07	52.39	47.51
		(4 July–17 July)	(17 July–7 August)	(7 August–22 August)	(22 August–12 September)	(12 September–5 October)
2013	m_f_ − m_0_ (mm)	19.04	−32.49	3.98	−0.88	17.04
	δ_f_m_f_ − δ_0_m_0_ (‰)	−73.20	350.94	−187.74	241.38	−288.66
	m_p_ (mm)	84.40	36.70	28.80	67.90	43.10
	δ_p_m_p_ (‰)	−767.07	−326.64	−215.17	−364.00	−426.85
	m_r_ (mm)	12.50	0.00	0.00	1.30	0.30
	δ_r_m_r_ (‰)	−123.57	0.00	0.00	−6.97	−2.97
	m_d_ (mm)	10.20	17.20	0.00	5.00	0.00
	δ_d_m_d_ (‰)	−90.06	−151.87	0.00	−44.15	0.00
	m_u_ (mm)	0.00	0.00	22.00	0.00	15.20
	δ_u_m_u_ (‰)	0.00	0.00	−194.25	0.00	−146.50
	δ_t_ (‰)	−5.98	−7.47	−4.05	−7.85	−5.01
	δ_e_ (‰)	−15.37	−15.82	−16.34	−15.94	−16.47
	E (mm)	23.96	16.46	2.62	7.86	6.67
	T (mm)	18.69	35.53	44.20	54.63	34.30

Note: m_0_, the soil water storage of original state (mm); m_f_, the soil water storage of final state (mm); m_p_, precipitation (mm); m_r_, runoff (mm); m_d_, deep percolation beyond the root zone (mm); m_u_, upward capillary rise into the root zone (mm); δ_0_, the δ^18^O of the soil water storage of original state (‰); δ_f_, the δ^18^O of the soil water storage of final state (‰); δ_p_, the δ^18^O of precipitation (‰); δ_u_, the δ^18^O of capillary rise (‰); δ_r_, the δ^18^O of surface runoff (‰); δ_d_, the δ^18^O of deep percolation (‰); δ_e_, the δ^18^O of evaporation (‰); δ_t_, the δ^18^O of transpiration (‰).

## Data Availability

The original contributions presented in this study are included in the article/Supplementary Materials. Further inquiries can be directed to the corresponding author.

## Supplementary Materials

The following supporting information can be downloaded at: https://www.mdpi.com/article/10.3390/plants15132059/s1.
