# Supplementary material for: Comparison of Isotope Mass Balance and AquaCrop Model in Evapotranspiration Partitioning in a Maize Field of North China
Source: Plants (Basel). 2026 Jul 2;15(13):2059. doi: 10.3390/plants15132059 (PMC13363973; doi:10.3390/plants15132059)
Supplement: Supplementary file 1 [file plants-15-02059-s001.zip › plants-4343582-supplementary.pdf]

Supplementary Table S1. Summary of input parameters for AquaCrop (V6.1) summer maize simulation at Shangzhuang Experimental Station (2011–2013)

Note: M=Measured (field/laboratory measurement); C=Calibrated with 2012 experimental data; D=FAO AquaCrop default value [40,53]; All simulations under rainfed condition (no artificial irrigation, no fertility or thermal stress).

[40] Steduto, P.; Hsiao, T.C.; Raes, D.; Fereres, E. AquaCrop—The FAO crop model to simulate yield response to water: I. Concepts and underlying principles. *Agron. J.* **2009**, *101*, 426–437.

[53] Hsiao, T.C.; Heng, L.; Steduto, P.; Rojas-Lara, B.; Raes, D.; Fereres, E. AquaCrop: The FAO crop model to simulate yield response to water: III. Parameterization and testing for maize. *Agron. J.* **2009**, *101*, 448–459.

Part 1: Crop core parameters

Parameter	Value	Unit	Source
Plant density	60606	plants·ha^{-1}	M
Maximum effective rooting depth	1.00	m	M
Base temperature	8	°C	D
Cut-off temperature	30	°C	D
Canopy transpiration coefficient (K_{cTr})	1.03	–	C
Normalized biomass water productivity (WP*)	30.70	g·m^{-2}	C
Reference harvest index (HI_0)	40	%	C
Leaf expansion upper threshold	0.14	–	D
Leaf expansion lower threshold	0.72	–	D
Stomatal stress upper threshold	0.69	–	D
Canopy senescence threshold	0.69	–	D
CN (SCS runoff curve number)	65	–	C
Readily evaporable water (REW)	8	mm	C

Part 2: Phenological schedule (DAP=days after planting)

Year	Planting Date	Emergence (DAP)	Max CC (DAP)	Senescence (DAP)	Maturity (DAP)	Source
2011	Jun 25	6	54	86	108	M
2012	Jun 23	6	54	84	105	M

Year	Planting Date	Emergence (DAP)	Max CC (DAP)	Senescence (DAP)	Maturity (DAP)	Source
2013	Jul 1	6	59	80	97	M

Part3 Layered soil parameters (0–100 cm, seven soil layers; θ_r residual water, θp field capacity, θ_s saturated water, K_s saturated hydraulic conductivity; a,n Van Genuchten parameters)

Soil depth (cm)	θ_r ($cm^3 \cdot cm^{-3}$)	θ_p ($cm^3 \cdot cm^{-3}$)	θ_s ($cm^3 \cdot cm^{-3}$)	a	n	K_s ($cm \cdot d^{-1}$)	Source
0–5	0.093	0.261	0.458	0.09	1.29	55.034	M
5–10	0.073	0.236	0.418	0.08	1.33	53.892	M
10–20	0.070	0.252	0.440	0.10	1.32	61.318	M
20–40	0.077	0.259	0.393	0.04	1.40	65.938	M
40–60	0.069	0.249	0.390	0.02	1.56	99.382	M
60–80	0.073	0.279	0.440	0.03	1.37	81.022	M
80–100	0.064	0.245	0.437	0.01	1.84	83.606	M

Part4 Climate & management setup

Item	Description	Source
Meteorological data	Daily Tmax/Tmin, precipitation, sunshine, RH from Beijing meteorological station; ET_0 calculated via FAO-PM equation	M
Irrigation	Rain-fed, zero supplementary irrigation throughout growing season	M
Fertility management	No nutrient stress, optimal soil nutrition	D
Initial soil water	Measured volumetric water content per layer at pre-sowing each year	M

[1] Wang J, Huang F, Li B. Quantitative analysis of yield and soil water balance for summer maize on the piedmont of the North China Plain using AquaCrop. *Front of Agr Sci Eng*. 2016;2(4):295-310.

[2] Hsiao TC, Heng L, Steduto P, Rojas-Lara B, Raes D, Fereres E. AquaCrop: The FAO crop model to simulate yield response to water: III. Parameterization and testing for maize. *Agronomy Journal*. 2009;101(3):448-459.

[3] Heng LK, Hsiao T, Evett S, Howell T, Steduto P. Validating the FAO AquaCrop model for irrigated and water deficient field maize. *Agronomy Journal*. 2009;101(3):488-498.

Supplementary Table S2. IMB-derived E, T, and T/ET with estimated uncertainties for each growth stage in 2012 and 2013.

Year	Date	E(mm)	T(mm)	T/ET(%)	±T/ET (%)*
2012	7/18-7/24	10.12	5.99	37.20	1.86
2012	7/24-8/14	18.43	50.47	73.25	3.66
2012	8/14-8/27	1.92	49.07	96.23	4.81
2012	8/27-9/12	2.20	52.39	95.97	4.80
2012	9/12-10/5	8.40	47.51	84.97	4.25
2013	7/18-7/24	23.96	18.69	43.82	2.19
2013	7/24-8/14	16.46	35.53	68.34	3.42
2013	8/14-8/27	2.62	44.20	94.40	4.72
2013	8/27-9/12	7.86	54.63	87.43	4.37
2013	9/12-10/5	6.67	34.30	83.73	4.19

Note: ±T/ET (%) is calculated as 5% of the T/ET value (relative uncertainty). E and T themselves are presented without error bars in Figure 4 due to the stacked-bar format limitation; their estimated relative uncertainty is ±10% (not shown for brevity).

Supplementary Table S3. Error statistics for AquaCrop compared to IMB for ET, E, and T in 2012 and 2013.

**Year**	**Variable**	**RMSE (mm)**	**MAE (mm)**	**Bias (mm)**	**RE (%)**	**NSE**	**d**
2012	ET	2.40	1.98	0.14	3.73	0.98	1.00
	E	3.56	2.69	-2.37	35.8	0.66	0.89
	T	5.20	4.35	2.51	12.0	0.91	0.98
2013	ET	3.20	2.76	1.86	5.47	0.83	0.97
	E	14.16	10.64	8.83	98.1	-2.39	0.71
	T	12.48	9.64	-6.97	34.8	-0.10	0.86

Notes: RMSE: Root Mean Square Error; MAE: Mean Absolute Error; Bias: Mean Bias Error (calculated as AquaCrop − IMB); RE: Relative Error (calculated as (Bias / Mean IMB value) × 100%); NSE: Nash–Sutcliffe Efficiency (1 = perfect agreement; values < 0 indicate model performance worse than using the mean IMB value); d: Willmott's Index

of Agreement (1 = perfect agreement). Positive bias indicates overestimation by AquaCrop, and negative bias indicates underestimation. All statistics were calculated based on n = 5 growth stages for each year.

Supplementary Text S1. Estimation of runoff (m_r), deep percolation (m_d), and upward capillary rise (m_u)

S1.1 Runoff (m_r)

Runoff was not measured directly. Instead, we used the runoff estimates generated by the AquaCrop model (Version 6.1), which employs the USDA-Soil Conservation Service (SCS) curve number method. The curve number was set to 78 for the loam/sandy loam soil under conventional tillage, following the default recommendation for the North China Plain. Daily runoff was calculated from daily rainfall and antecedent soil moisture conditions as simulated by AquaCrop. The cumulative runoff over each growth stage is reported in Table 1 of the main manuscript.

S1.2 Deep percolation (m_d)

Deep percolation was also taken from AquaCrop model outputs. In AquaCrop, percolation occurs when the soil water content in a layer exceeds field capacity; the excess water drains to the next layer. Water that leaves the bottom of the root zone (100 cm) is considered deep percolation. The cumulative percolation over each growth stage is listed in Table 1. No independent measurements were available; therefore, the uncertainty associated with percolation (±10–15%) was included in the sensitivity analysis (Section 2.4 of the main manuscript).

S1.3 Upward capillary rise (m_u)

Capillary rise was not measured directly. It was estimated as the residual of the water balance equation (Equation 4 in the main text) after all other components (precipitation, irrigation, runoff, deep percolation, soil water storage change, evaporation, and transpiration) were known. Specifically, rearranging Equation (4):

$$m_u = (m_f - m_0) + m_r + m_d + m_e + m_t - m_p - m_i$$

In periods where the calculated residual was positive, it was interpreted as upward capillary rise; when negative, it was set to zero (indicating no net upward flow). This residual method is standard in field water balance studies when groundwater contributions are expected to be small or when direct measurements are unavailable [36,48]. The estimated capillary rise values for each growth stage are presented in Table 1.

The uncertainties in these derived components were propagated into the final T/ET uncertainty, as described in Section 2.4 of the main manuscript.
